# Use of a Molecular Decoy to Segregate Transport from Antigenicity in the FrpB Iron Transporter from *Neisseria meningitidis*

**DOI:** 10.1371/journal.pone.0056746

**Published:** 2013-02-15

**Authors:** Muhammad Saleem, Stephen M. Prince, Stephen E. J. Rigby, Muhammad Imran, Hema Patel, Hannah Chan, Holly Sanders, Martin C. J. Maiden, Ian M. Feavers, Jeremy P. Derrick

**Affiliations:** 1 Michael Smith Building, Faculty of Life Sciences, University of Manchester, Oxford Road, Manchester, United Kingdom; 2 Manchester Institute of Biotechnology, University of Manchester, 131 Princess Street, Manchester, United Kingdom; 3 National Institute for Biological Standards and Control, Health Protection Agency, Blanche Lane, South Mimms, Potters Bar, Hertfordshire, United Kingdom; 4 Department of Zoology, University of Oxford, South Parks Road, Oxford, United Kingdom; Monash University, Australia

## Abstract

FrpB is an outer membrane transporter from *Neisseria meningitidis*, the causative agent of meningococcal meningitis. It is a member of the TonB-dependent transporter (TBDT) family and is responsible for iron uptake into the periplasm. FrpB is subject to a high degree of antigenic variation, principally through a region of hypervariable sequence exposed at the cell surface. From the crystal structures of two FrpB antigenic variants, we identify a bound ferric ion within the structure which induces structural changes on binding which are consistent with it being the transported substrate. Binding experiments, followed by elemental analysis, verified that FrpB binds Fe^3+^ with high affinity. EPR spectra of the bound Fe^3+^ ion confirmed that its chemical environment was consistent with that observed in the crystal structure. Fe^3+^ binding was reduced or abolished on mutation of the Fe^3+^-chelating residues. FrpB orthologs were identified in other Gram-negative bacteria which showed absolute conservation of the coordinating residues, suggesting the existence of a specific TBDT sub-family dedicated to the transport of Fe^3+^. The region of antigenic hypervariability lies in a separate, external sub-domain, whose structure is conserved in both the F3-3 and F5-1 variants, despite their sequence divergence. We conclude that the antigenic sub-domain has arisen separately as a result of immune selection pressure to distract the immune response from the primary transport function. This would enable FrpB to function as a transporter independently of antibody binding, by using the antigenic sub-domain as a ‘molecular decoy’ to distract immune surveillance.

## Introduction

*Neisseria meningitidis* is the causative agent of meningococcal disease, a serious global public health problem. Development of a vaccine against the serogroup B form of the disease has focused on the outer membrane proteins (OMPs), notably the porin protein PorA and, more recently, factor H binding protein [1]. It is also known that other OMPs contribute to protective immunity, including the FrpB iron transporter (also known as FetA), which has been a component of several outer membrane vesicle-based vaccines [2]. The inclusion of OMPs in vaccines is complicated by their extensive sequence variation, which is located within certain surface-exposed regions of these proteins. Consequently, specific OMP variants provide protective immunity against only a narrow range of strains expressing those same variants. This is a poorly understood problem at the level of protein structure, despite its major implications for prophylaxis.

FrpB is predicted, on the basis of sequence similarity, to belong to the large family of TonB-dependent outer membrane transporters (TBDTs, which are responsible for the uptake of iron, heme, vitamins and other substrates into the periplasm, powered by interaction with the inner membrane proteins TonB, ExbB and ExbD [3], [4], [5], [6]. TBDTs also play a role in the transport of some colicins across the outer membrane [7], [8]. Canonical TBDT structures consist of a 22-stranded β-barrel with an N-terminal α/β ‘plug’, which fills the interior and plays a role in substrate recognition [6], [9]. To date, crystal structures have been described for iron-siderophore, heme and vitamin B12 TBDTs [3], [10], although there is now good evidence that substrate specificities for other TBDTs extend wider [11]. With the determination of over a dozen TBDT crystal structures, several conserved motifs have become apparent, distributed across the plug domain and the residues which line the interior of the β-barrel [6]. There is much wider variation in the external loop regions, which play a critical role in capturing the transported substrate. A vital feature of any TBDT is the TonB box, a short, partly conserved sequence of residues found at the N-terminus. It forms the region of the transporter which interacts with a periplasmic domain from TonB, through a short β-β strand pairing [12], [13]. Despite intensive structural and biophysical investigation over the last 15 years, however, the precise mechanism of substrate transport through the barrel lumen is unclear. Studies by EPR have shown that substrate binding induces an unfolding of the TonB box residues [14], [15]; this must somehow perturb or displace the plug domain to allow the passage of substrate. To complicate matters further, experiments using fluorophores to label specific cysteine residues in the plug domain have indicated that there are significant differences between different transporters [8], [16], [17].

The importance of iron uptake to pathogenic *Neisseria* is well established, with multiple uptake systems which can extract iron from transferrin, lactoferrin and heme [18]. Each of these sources of iron is mediated by a separate TBDT, and the structural basis for the release of iron from human transferrin by TbpA has recently been described [19]. In contrast to these specialized iron/heme transporters, the specificity of FrpB is less clear. Expression of FrpB is induced under iron-limiting conditions, under control of the Fur ferric iron regulator, acting through the AraC-like MpeR protein [20], [21]. Binding studies carried out in whole *N. gonorrhoeae* have shown that FrpB has a relatively weak affinity (15 µM) for ferric enterobactin [22], and more recent work has shown that it can transport iron derived from at least four different catecholate-type siderophores [21]. The current view of FrpB substrate transport specificity is therefore that it is broader in its substrate specificities than other TBDTs, and potentially capable of transporting a range of different substrates.

Much less attention has been paid to the role of TBDTs as antigens, however, as opposed to their role as transporters. For example, FrpB is capable of eliciting the production of bactericidal antibodies against meningococci [23]. Sequence variation in FrpB from different meningococcal strains is mainly concentrated into a single region of approximately 40 residues, which is predicted to lie in an exposed location on the outer surface of the bacterium [24]. Epidemiological studies have shown that some antigenic types are highly persistent over time: this behavior is reproduced in mathematical models which assume a strong immune selection pressure, resulting in the emergence of different variants [25], [26], [27], [28], [29]. The *N. meningitidis* genome is subject to high rates of recombination, but it is apparent that only a few lineages are disease-causing and the associated clonal complexes retain stable combinations of antigenic alleles [30].

The structural consequences for antigenic variability within TBDTs, and its ramifications for recognition of transported substrate, remain unexplored. Here we report the determination of the crystal structures of two major antigenic variants of *N. meningitidis* FrpB. We show that both adopt the same structural motif which protrudes well above the predicted location of the outer membrane. In addition, we show that FrpB binds Fe^3+^ with high affinity, and that this binding site is separate from the antigenic region. Our results suggest that immune selection pressure has resulted in the emergence of a surface antigenic motif which is apparently able to camouflage the iron binding site. The process of selection of different antigenic variants within the bacterial population can therefore proceed without impairing the core transport function of FrpB.

## Materials and Methods

### Protein expression, refolding and purification

Expression, purification and crystallization of FrpB F3-3 and F5-1 (Fe-bound form) were carried out as described previously [31]. The sequences of both expression open reading frames were determined by DNA sequencing. To introduce iron, a crystal of FrpB F5-1 was soaked in 30 mM di-ethylene glycol, 30 mM tri-ethylene glycol, 30 mM tetra-ethylene glycol, 30 mM penta-ethylene glycol, 45 mM imidazole, 20% (w/v) PEGMME550, 10% (w/v) PEG 20,000 and 56 mM MES/NaOH (pH 6.5) containing 10 mM FeSO_4_, for 10 minutes, before back-soaking in the same solution, but without FeSO_4_, for a further 10 minutes. The crystal was then frozen in liquid nitrogen for data collection, as described previously [31]. To form crystals of FrpB F5-1 without Fe bound, FrpB (9.5 mg/ml) in 50 mM TrisHCl (pH 7.9), 200 mM NaCl, 0.5% (v/v) C_8_E_5_ and 50 mM imidazole was added to 0.23 mM enterobactin final concentration (E3910, Sigma). 200 nl of the FrpB-enterobactin solution was then added to 200 nl of crystallization reservoir solution, containing 20 mM Na L-glutamate, 20 mM D, L-alanine, 20 mM glycine, 20 mM lysine (racemic), 20 mM serine (racemic), 40 mM imidazole, 24% (w/v) PEGMME550, 12% (w/v) PEG 20,000 and 61 mM MES/NaOH (pH 6.5). Crystals were allowed to grow at 20°C. For data collection, the crystal was washed in crystallization reservoir solution plus 0.5% pentaoxyethylene octyl ether, prior to freezing. The addition of enterobactin to the crystallization reservoir solution helped to remove any iron contamination in the medium.

### Site-directed mutagenesis of FrpB F3-3 and F5-1

Mutagenesis was carried out using the Quikchange Lightening mutagenesis kit from Agilent Biosciences, according to the manufacturer's protocol. For mutation of histidine 133 to alanine, the following primers were used: CCGACAGCCAAATCCTTTAC**GCT**CAAGGCAGATTTATTGTCG and CGACAATAAATCTGCCTTG**AGC**GTAAAGGATTTGGCTGTCGG. For mutation of tyrosine 347 to phenylalanine, the following primers were used: CGATGACAGCGGCACCGGC**TTC**GCAGGCAATGTAAAAGGC and GCCTTTTACATTGCCTGC**GAA**GCCGGTGCCGCTGTCATCG (modified bases highlighted in bold).

### Structure determination and refinement

Data were processed using XDS [32]; data reduction and refinement statistics are summarized in Table 1. The CCP4 program suite [33] was used for structure solution of FrpB F3-3. The best model for molecular replacement was identified by the program BALBES [34] (database date Jun 1 2011) as 3FHH, the ShuA heme transporter from *Shigella dysenteriae* [35], with a sequence identity of 20.7% over 616 aligned residues. The program CHAINSAW [36] was used to modify 3FHH to an FrpB-template structure using CLUSTALW [37] sequence alignment so that the maximal number of common atoms were preserved [38]. This search model was used with the program PHASER [39] in automatic molecular replacement mode to search for z = 3 copies of the FrpB-template in the asymmetric unit, the translation function Z-score for the final component of the solution was 12.1 and the log-likelihood gain 305 (in a control run searching ror z = 2 copies PHASER placed two of the three copies of FrpB-template in the z = 3 solution). The resulting solution showed a compelling packing arrangement with the three copies of the FrpB-template oriented in a parallel fashion about the non-crystallographic symmetry (NCS) 3-fold axis observed in the data [31].

**Table 1:** Data collection and refinement statistics.

FrpB variant	F3-3	F5-1 (Fe)		F5-1 (apo)
Space group	*P 2_1_ 2_1_ 2_1_*	*C2*		*C2*
Unit cell parameters	a = 85.3 Å, b = 104.6 Å,c = 269.1 Å	a = 176.0 Å, b = 78.7 Å,c = 73.4 Å, β = 97.2°		a = 175.2 Å, b = 79.1 Å,c = 74.7 Å, β = 97.6°
X-ray source and wavelength (Å)	DLS^b^ I24 (0.978)	DLS I24 (0.969)	DLS I24 (1.740)	DLS I24 (0.969)
Resolution range (Å)	134–2.40 (2.46-2.40)^a^	36–2.60 (2.67-2.60)	40-5.0 (5.13-5.0)	37-2.32 (2.38-2.32)
Multiplicity	3.8 (2.2)	2.6 (2.6)	3.2 (3.1)	2.2 (2.2)
Significance (<I>/sd)	10.2 (2.3)	12.7 (2.2)	11.3 (7.3)	9.8 (2.0)
No. unique reflections	86,235	29,989	27,289	43,114
Completeness (%)	90.7 (83.1)	97.4 (98.9)	99.2 (99.4)	98.2 (98.8)
*R_merge_* (%)^c^	10.8 (37.9)	5.5 (51.7)	9.3 (14.6)	5.0 (29.8)
**Refinement Statistics**				
R_cryst_	24.2	22.7		23.3
R_free_ (5% data)	29.6	27.8		28.1
Nonhydrogen atoms				
All	15,985	5,222		5,182
Water	328	44		80
Mean overall B (Å^2^)	22.5	48.9		45.1
RMSD from ideal values				
Bond distance (Å)	0.024	0.024		0.017
Bond angle (degrees)	2.1	2.2		1.9

a values in parentheses refer to the outer resolution shell.

b Diamond Light Source.

c *R_merge_* = Σ_hkl_Σ_sym_|I−<I>|/Σ_hkl_I.

A slabbed omit map was subsequently calculated and back transformed to obtain a less biased phase set [40]. The 3-fold NCS rotation axis vector was determined from the molecular replacement solution (direction cosines relative to orthogonal coordinates: −0.941, −0.019, 0.330). Coordinates within a thickness of 4 Å were omitted in slabs perpendicular to the 3-fold NCS axis and a 2F_o_-2F_c_ difference map was calculated using the programs SFALL, RSTATS and FFT. This procedure was repeated for 19 sections comprising the length of the FrpB-template trimer along the NCS axis. The resulting maps were combined by addition in the program OVERLAPMAP, and a phase set and figure of merit was calculated through back-transformation of this omit map using SFALL, RSTATS and SIGMAA [41]. These phases along with a mask generated from the FrpB-template (programs LSQKAB [42] and NCSMASK) were submitted as a starting point for density modification using the program DM [43] implementing solvent flattening (solvent content 51%), histogram matching and NCS symmetry averaging. The resulting map clearly showed electron density for trans-membrane β strands, and the central plug domain.

The structure of the FrpB F3-3 variant was built manually using COOT [44], with some loop building carried out using BUCCANEER, from the CCP4 suite [33]. Refinement, which included non-crystallographic symmetry constraints between the three chains of the trimer, was carried out using REFMAC [45]. Residues 1–59, 401–416, 568–573 and 673–675 were omitted from some or all chains due to poor electron density. The final model contained two pentaoxyethylene octyl ether (C_8_E_5_) detergent molecules, located between the membrane-spanning parts of the adjacent β-barrels, and a single glutamate molecule, derived from the crystallization medium [31].

A monomer from the FrpB F3-3 structure (chain A) was used to solve the structure of the F5-1 (Fe) variant, in the C2 crystal form (Table 1), by molecular replacement implemented using MOLREP [46]. The hypervariable loop region was omitted from the model during initial refinement and subsequently built manually with COOT. In addition to the bound Fe atom, electron density maps showed evidence for an imidazole molecule, presumably derived from the crystallization medium where it is present at 50 mM, chelating the Fe. The structure of the F5-1 apoprotein form (designated F5-1 (apo) in Table 1) was solved using the structure of the F5-1 (Fe) variant by molecular replacement using MOLREP. Following initial refinement using REFMAC, differences between the structures became apparent in two external loop regions and the immediate N-terminus of the plug domain. These regions were subsequently rebuild manually using COOT and the structure subjected to torsional refinement, as implemented within CNS [47], [48]. Some linear density was observed on the external surface of the beta barrel which probably originated from the C_8_E_5_ detergent used during crystallization, but was it too small to model a complete detergent molecule.

Stereochemical parameters for all structures were examined using PROCHECK [49], and were within or better than the tolerance limits expected for each structure at the resolution limits given in Table 1. Figures were prepared using CCP4MG [50].

### Size exclusion chromatography (SEC) and binding experiments

SEC was carried out as described previously [31], using a Superdex 200 10/30 gel filtration column (GE Healthcare). For Fe binding studies, Fe-citrate or Fe-enterobactin were prepared by pre-incubation, before addition to FrpB F3-3. The Fe-citrate complex was made by mixing sodium citrate and FeCl_3_ in a 5∶1 molar ratio. This solution was then combined with 16.5 nmol (33 µM) of FrpB F3-3 such that the Fe-citrate complex was present in a 30-fold molar excess (i.e. 990 µM final concentration) and incubated for 1 hour at 20°C, followed by injection on to the Superdex 200 10/30 column. For the Fe-enterobactin binding experiment, the Fe-enterobactin complex was formed by mixing enterobactin and FeCl_3_ in a 2∶1 molar ratio (so that all Fe^3+^ was in the enterobactin-bound state). This solution was then mixed with 16.5 nmol (33 µM) of FrpB F3-3 such that the Fe-enterobactin complex was present in a 10-fold molar excess (i.e. 330 µM). Following incubation for 1 hour at 20°C, the solution was injected on to the Superdex 200 10/30 column. Control samples repeated the procedure using the same quantity and concentration of FrpB F3-3, but with no potential ligand added. 1 ml of each peak fraction was acidified with 2% nitric acid, the FrpB F3-3 precipitate removed by centrifugation at 7,000 g for 10 min at 15°C, the supernatant recovered and filtered through a 0.22 µm filter before ICP-MS analysis.

### EPR

FrpB samples for EPR were purified as described previously for crystallization [31], except that the SEC step was carried out using a Hiload 16/60 Superdex 200 column (GE Healthcare), equilibrated in 20 mM TrisHCl (pH7.4), 150 mM NaCl and 0.1% (v/v) lauryldimethylamine-oxide (LDAO). For the studies with enterobactin, FrpB was concentrated to 130 µM; the enterobactin∶Fe^3+^ complex was prepared by mixing enterobactin and FeCl_3_ in a 2∶1 ratio, before addition to FrpB such that the final Fe concentration was 65 µM. For the control sample (enterobactin∶Fe in buffer), the same procedure was carried out but without addition of FrpB. For the FrpB∶Fe^3+^ complex, 150 µM FrpB was added to 100 µM FeCl_3_ and the complex dialysed against 20 mM TrisHCl (pH7.4), 150 mM NaCl and 0.1% (v/v) LDAO for 36 hours at 20°C. For the Fe^3+^/Cu^2+^ competition experiment, 150 µM FrpB was added to 100 µM Cu_2_SO_4_ and then dialysed against 20 mM TrisHCl (pH7.4), 150 mM NaCl and 0.1% (v/v) LDAO for 36 hours at 20°C. FeCl_3_ was then added to various concentrations before mixing and freezing. Continuous wave EPR spectra were collected at X-band (approximately 9.4 GHz) using a Bruker ELEXSYS E500/580 EPR spectrometer equipped with Bruker ‘Super High Q’ (SHQE) resonator. Sample temperature control was governed using an Oxford Instruments ESR900 cryostat coupled to an ITC503 temperature controller. Experimental parameters: microwave power, 0.1 mW; field modulation frequency 100 KHz; field modulation amplitude 7G; temperature 10 K.

### Antibody production

FrpB-polyclonal sera was obtained from immunisation of ten, female NIH/OlaHsd mice (18–22 g, Harlan Laboratories, Indiana, USA) with two doses of recombinant FrpB F3-3 protein (10 µg/dose with 1∶66 w/w protein∶Al(OH)_3_). Mice were immunised subcutaneously at days 0 and 21, and bled on day 35. Sera from all ten mice were pooled before use. Mouse immunisations were carried out in accordance with the Animals (Scientific Procedures) Act 1986 (UK), under project licence (PPL) number 80/2157. Procedures carried out under this licence were approved by the NIBSC ethics committee. The severity of the procedure was classified as mild and all appropriate measures were taken to minimize suffering. Final bleeds were taken by cardiac puncture with terminal anaesthesia. The FrpB F3-3 monoclonal antibody was produced by ABDSerotec (Oxford, UK) using immunization of Balb/c mice with two doses of 50 µg each recombinant FrpB F3-3 protein with Sigma Adjuvant System (Sigma Aldrich). Hybridomas were screened for reactivity with meningococcal whole cells expressing the F3-3 protein, with negative screening against cells from a strain not expressing FrpB. Hybridomas were further screened against multiple FrpB variants for specificity against F3-3. Following selection, the hybridoma was grown for antibody production by Cancer Research UK (Potters Bar, UK). Culture supernatant containing the antibody was extracted for use.

### Slot blot

All samples were diluted in TBS (0.025 M Tris/137 mM NaCl (adjusted to pH 7.6 with hydrochloric acid) to a final concentration of 2 µg/ml total protein. Whole cells of *Neisseria meningitidis* were extracted from overnight growth on Colombia Blood agar plates with 5% Defibrinated Horse Blood (both Oxoid, Cambridge, UK) and heat killed at 56°C for 30 minutes prior to dilution. 900 ng of each sample was transferred to Hybond-C nitrocellulose membrane (GE Healthcare, Buckinghamshire, UK) using Bio-Dot SF Apparatus (BioRad Laboratories, California, USA), according to manufacturer's instructions. After transfer, the membrane was incubated in dilution buffer followed by primary antibody: FrpB F3-3 monoclonal antibody at a 1∶100 dilution; FrpB polyclonal serum at a 1∶400 dilution. The dilution buffer used was TBS/5% (w/v) Marvel milk powder (Premier International foods, Lincolnshire, UK). All incubations were at room temperature for one hour. Before and after addition of secondary antibody (Anti-Mouse IgG-HRP conjugate, Sigma Aldrich, at a 1∶200 dilution), the membrane was washed in 3×five minutes changes of TBS. The membrane was stained using Supersignal West Pico Chemiluminescent substrate (Pierce, Illinois, USA), according to manufacturer's instructions, and imaged using a Gel Logic 1500 Imaging System (Carestream, New York, USA) set to record luminescence over 3×5 minute exposures.

### Enzyme-linked Immunosorbent Assay

Proteins samples were diluted in PBS (137 mM NaCl/2.7 mM KCl/10 mM Na_2_HPO_4_/1.8 mM KH_2_PO_4_ (pH adjusted to 7.4 with hydrochloric acid) to 0.5 µg/ml and 100 µl was added to each well of a NUNC flat-bottomed 96-well microtitre plate (Thermo Fisher, Massachusetts, USA). Plates were incubated overnight at 4°C. All further dilutions were in PBS/0.5% bovine serum albumin/0.01% Tween-20, and incubations were carried out at room temperature. Plates were washed three times in 0.025% v/v Tween-20 before addition of 100 µl/well monoclonal antibody at a starting dilution of 1∶500. A 1∶3 dilution series was made down the plate. Following incubation for one hour, plates were washed again before addition of 100 µl/well Anti-Mouse IgG-HRP conjugate (Sigma Aldrich) and further incubation for one hour. Plates were then washed three times, before addition of 100 µl/well of tetramethylbenzidine ELISA substrate (Universal Biologicals, Cambridgeshire, UK). After ten minutes, 100 µl/well of 1 M sulphuric acid was added and absorbance was read at 450 nm using a Multiskan MS plate reader (Labsystems, Helsinki, Finland).

### Accession codes

The structures of FrpB variants F3-3, F5-1 (Fe) and F5-1 (apo) have been deposited in the Protein Data Bank with accession codes 4AIP, 4AIQ and 4B7O respectively.

## Results

### The Structure of FrpB and Identification of the Fe Binding site

**Figure 1:**
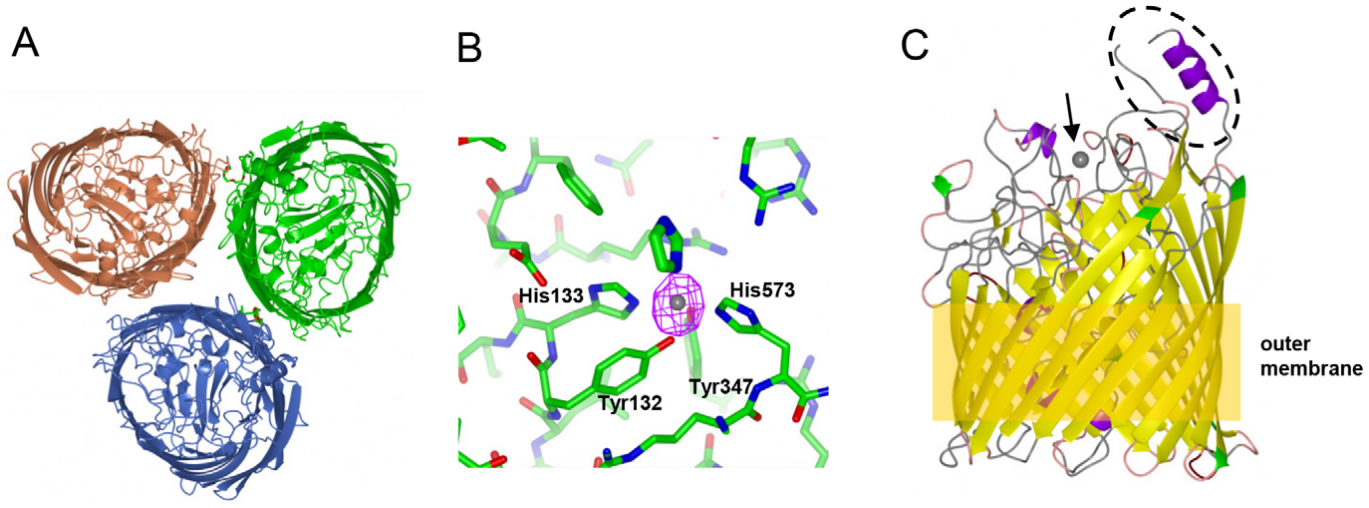
Structure and iron recognition by FrpB. (A) FrpB F3-3 variant trimer, viewed from above. (B) Detail of the Fe binding site in FrpB F5-1; Fe coordinating residues are marked. The anomalous difference map is shown in purple, contoured at 8σ, superimposed on the Fe atom. (C) Ribbon plot of FrpB F5-1, coloured by secondary structure, indicating the relative positions of Fe (arrowed), the helix-loop HR region (circled) and the outer membrane.

We obtained crystals of two different sequence variants of FrpB, termed F3-3 and F5-1 [24]; these variants differ in sequence predominantly within a region of 30–40 residues which is predicted to be surface-exposed. We initially solved the structure of the F3-3 variant by molecular replacement using the *Shigella* ShuA heme transporter structure as a search model [35] (Table 1). Our previous work established that FrpB elutes from a size exclusion column (SEC) in two peaks: one corresponds to the expected mass for a monomer and the other, higher molecular mass peak, was attributed to trimers or possibly other higher molecular mass species or aggregates [31]. Analysis of the self-rotation function suggested that FrpB F3-3 forms a trimer in the asymmetric unit. Solution of the structure verified that this is indeed the case, with three FrpB F3-3 monomers arranged in a similar manner to that commonly found in porin proteins [51] (Figure 1A). FrpB adopts a classical TBDT structure, with a 22-stranded β-barrel and a plug domain at the N-terminus, located within the lumen of the barrel. Calculation of the interface area between FrpB monomers using the PISA server [52] (http://www.ebi.ac.uk/pdbe/prot_int/pistart.html) gave a mean value of 897 Å^2^, suggesting that this interaction might persist in the detergent-solubilized state. The use of non-crystallographic symmetry averaging was valuable in obtaining electron density of sufficient quality to allow building of the loop regions of FrpB which differed from ShuA. This enabled the complete building of nine external loop regions outside the β-barrel, leaving two incomplete due to weak or absent electron density: these were part of the region of sequence hypervariability (residues 401 to 416) and external loop 8 (residues 568–573). The FrpB F3-3 coordinates were then used to solve the structure of the FrpB F5-1 variant, which is a monoclinic crystal form with a monomer in the asymmetric unit (Table 1). Interestingly, FrpB F5-1 did not form a trimer in a similar fashion to F3-3: the hypervariable loop region forms a crystal contact with an adjacent monomer in the lattice, which may explain why the monomeric form is preferred for this particular sequence variant. It should also be noted that we consistently failed to obtain satisfactory crystals of either FrpB variant if we used the higher molecular weight species from SEC: only monomeric FrpB F3-3 or F5-1 were able to produce good crystals. One reason could be that the first peak to elute from SEC contains some misfolded or aggregated material which inhibits crystallization.

Solution of the FrpB F5-1 monoclinic crystal structure revealed the presence of a peak of high electron density, characteristic of a bound ion, coordinated by residues from the barrel and plug domain. This observation led us to repeat the data collection for an FrpB F5-1 crystal, but presoaking with iron before freezing. Data were then collected at high and low energies, the latter tuned to measure the anomalous diffraction from the bound iron atom (Table 1). The highest peak in the anomalous difference map, at 11.2σ, coincided with the bound ion (Figure 1b). The next highest peak in the map was at 4.5σ, demonstrating that this is a unique and specific binding site. The iron-binding site lies within the central part of the β-barrel and is formed by Tyr132 and His133 from the plug domain, and Tyr347 and His573 from external loops 4 and 8, which extend from the ends of the antiparallel strands in the barrel. A fifth coordination site is provided by an imidazole molecule, which was present in the crystallization medium. The coordination complex is based on an octahedral geometry, and is similar in that respect to the binding of the ferric ion by the periplasmic Fbp binding protein from *H. influenzae* and *N. gonorrhoeae* [53], [54]. In Fbp, however, the iron is bound by side chains from a histidine, glutamate and two tyrosine residues, with other coordination sites occupied by phosphate and water molecules. FrpB therefore lacks a coordinating carboxylate-containing side chain but has effectively substituted this with a second histidine residue.

**Figure 2:**
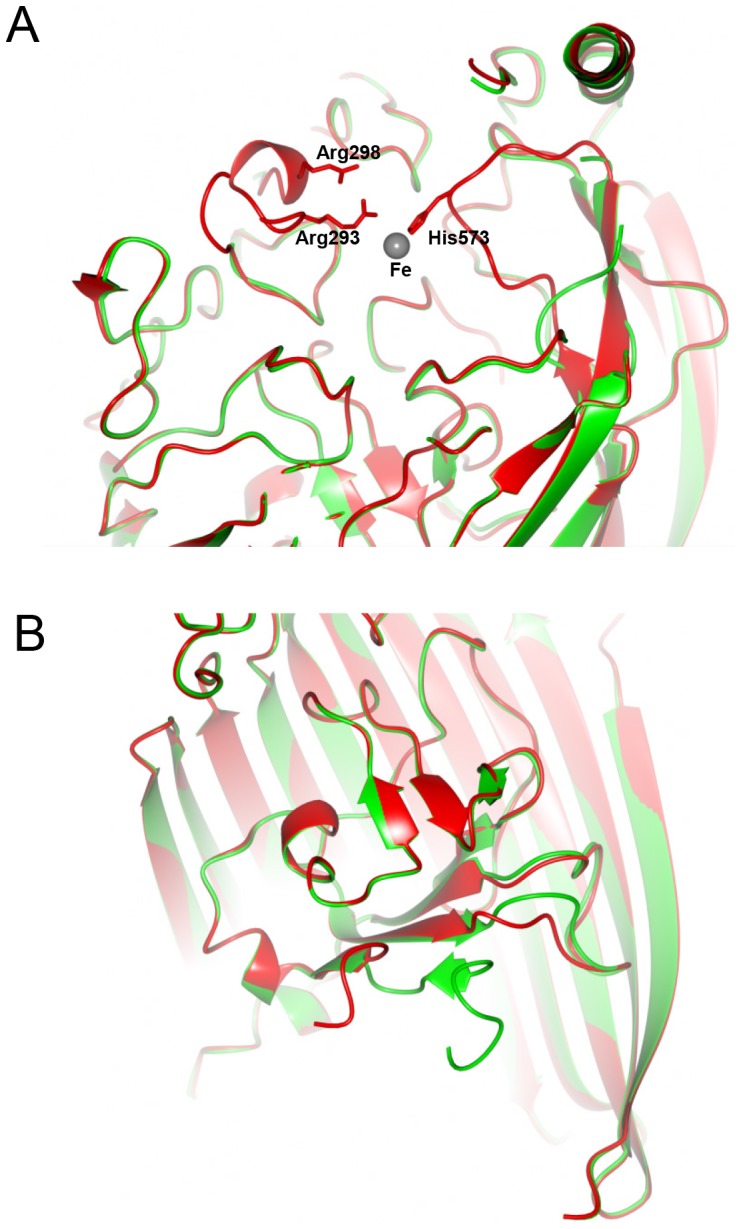
Structural changes on binding of Fe to FrpB F5-1. (A) External loop movement during Fe recognition. The structure of FrpB F5-1 with Fe bound is shown in red, superimposed on the apoprotein form in green. The locations of Arg293, Arg298 and His573 are shown relative to the bound Fe atom. (B) Change in the conformation of the N-terminus as a result of Fe binding. Part of the β-barrel has been removed, for clarity. Colors used are as for part (A).

A comparison of the FrpB F5-1 variant structures in the Fe-bound and unbound (apoprotein) states revealed two specific structural changes which apparently occurred on binding of Fe. First, portions of loops 3 and 8 were absent from electron density maps for the apoprotein form, but were structured in the Fe-bound state (Figure 2A). One of the Fe-coordinating residues, His573, is located at the tip of loop 8. Arg293 and Arg298 from loop 3 are not involved in directly chelating the Fe atom, but are located close to it; their conservation in FrpB homologs suggests that they could also play an important role in recognition of transported substrates (discussed further below). Movement of these loop regions therefore suggests an induced fit model for Fe binding. A second point of difference concerns the N-terminus: electron density for the apoprotein form starts at Asp61, 2–3 residues after the predicted location of the TonB box sequence (the site for binding of the inner membrane TonB protein). In the case of the Fe-bound FrpB structure, however, traceable electron density did not start until Ala67, and residues 67–71 adopt a different conformation (Figure 2B). These observations accord with work conducted on other TBDTs: the external loops in FecA are involved in an ‘induced fit’ type mechanism when binding ferric citrate [55] and there is evidence from EPR studies that the TonB box region becomes more disordered on binding of transported substrate [14]. The fact that structural changes in FrpB on binding of iron mirror those found in other TBDTs therefore provides additional evidence that iron is indeed a substrate for this transporter.

### Measurement of Fe Binding to FrpB and Analysis by EPR

**Figure 3:**
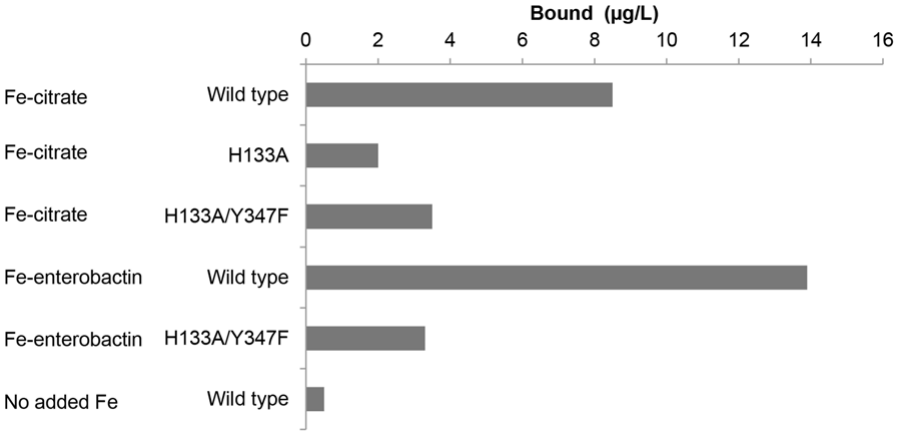
Elemental analysis of SEC peaks by ICP-MS. Values given are means of triplicates and relate to measured iron content by ICP-MS in the SEC fraction collected at 11–13 ml, corresponding to the elution position of the FrpB monomer. 33 µM FrpB F3-3 was mixed with either 990 µM Fe-citrate or 330 µM Fe-enterobactin, before separation by SEC.

To complement the crystallographic studies, we carried out a series of experiments to investigate the binding of iron to FrpB. Fe^3+^, as iron (III) citrate, was added to FrpB, and the sample fractionated by SEC as described previously [31]. Peaks containing either Fe-FrpB (bound fraction) or iron (III) citrate alone (unbound fraction) were collected and analyzed by inductively coupled plasma mass spectrometry (ICP-MS) for iron content (Figure 3). FrpB which had been exposed to iron (III) citrate consistently showed higher iron content compared with controls where no iron had been added to the protein. Further, mutation of His133 to Ala effectively abolishes iron content within the FrpB SEC peak; this was also the case for a H133A/Y347F mutation. Previous work in *N. gonorrhoeae* has shown that FrpB can use Fe^3+^-enterobactin as a transported substrate [22]; we were also able to demonstrate that the Fe content of the FrpB fraction increased following exposure to ferric enterobactin, and that this was much reduced for the H133A/Y347F double mutant (Figure 3). Such an observation would therefore be consistent with the binding of Fe^3+^-enterobactin to FrpB.

**Figure 4:**
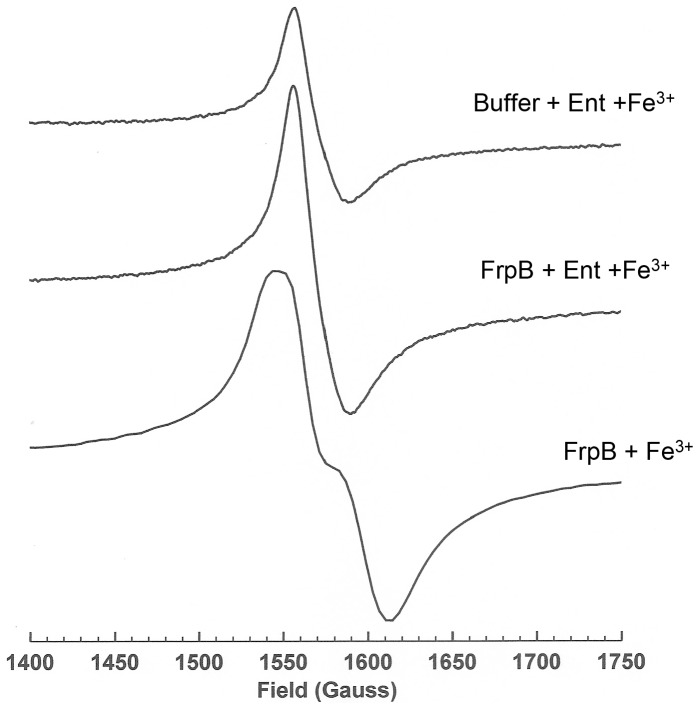
EPR spectra of Fe^3+^ and Fe^3+^–enterobactin bound to FrpB. EPR spectra are shown of 130 µM ferric-enterobactin (top), 130 µM ferric-enterobactin plus 130 µM FrpB F3-3 (middle) and 150 µM FrpB F3-3 which had been pre-incubated with Fe^3+^, followed by dialysis to remove unbound iron (bottom).

The binding of Fe^3+^ and Fe^3+^-enterobactin to FrpB was examined further by EPR (Figure 4). The Fe^3+^-enterobactin complex in buffer gave a sharp signal at *g* = 4.25, consistent with previous observations [56]. Addition of FrpB increased the amplitude of the signal but was clearly distinguishable from the spectrum of Fe^3+^ bound directly to FrpB (the lower spectrum in Figure 4), which was broader and split into two components at the centre. This latter spectrum is typical of a mononuclear Fe^3+^ site in which no ligand distance or interaction dominates and thus confirms the presence of a single, high affinity Fe^3+^ binding site within the protein.

Given the similarities between Fe^3+^ and Cu^2+^ binding sites in other proteins, and the evidence for Cu^2+^ transport by other TBDTs [11], Cu^2+^ binding to FrpB was also examined by EPR. FrpB was pre-incubated with Cu^2+^ and increasing concentrations of Fe^3+^, and a series of EPR spectra collected (Figure S1). Both FrpB variants showed evidence for a single Cu^2+^ site (A_∥_ = 192G). However, increasing concentrations of Fe^3+^ did not alter the Cu^2+^ signal for either variant. Repetition of the experiment with the FrpB H133A mutant showed that the Cu^2+^ signal was unimpaired by the mutation, although the Fe^3+^ signal was reduced (Figure S2). We conclude that the EPR results support the identification of the Fe^3+^ site from the FrpB crystal structure, but that Cu^2+^ binding is to a different location within the FrpB structure. Attempts were made to soak the F5-1 monoclinic crystals with cryoprotectant solution containing CuSO_4_, but they led to cracking and a serious deterioration in diffraction quality, precluding the collection of useful crystallographic data.

### Identification of TBDTs from other bacteria which are related to FrpB

We were able to identify FrpB orthologs from other Gram-negative bacteria, including *Pasteurella* and *Haemophilus*, where the coordinating residues for the Fe^3+^ ion in the structure are absolutely conserved (Figure S3). Sequences were most variable around His573, probably because the lengths of external loop 8 differ between orthologs. Nevertheless, all four coordinating residues were absolutely conserved and therefore effectively define a subfamily of TBDTs which appear to be specific for transporting Fe^3+^. In addition, although not directly interacting with the bound Fe^3+^, there are several Arg residues (282, 293, 298 & 725) which lie close to the iron binding site and are also highly conserved in sequence alignments. They contribute to a highly basic environment which is formed around the Fe^3+^ atom on binding: whether these residues are important for Fe^3+^ binding, or play a role in the recognition of more complex ligands such as Fe-enterobactin, is unclear at present. It is noteworthy that the principal region of antigenic hypervariability in *N. meningitidis* FrpB is completely absent from the other exemplar members of the TBDT subfamily. For other FrpB orthologs, this section of the structure runs from Asn396 directly to Tyr435, effectively pruning the antigen loop. We infer that this part of the structure plays no part in the recognition and transport of Fe^3+^.

**Figure 5:**
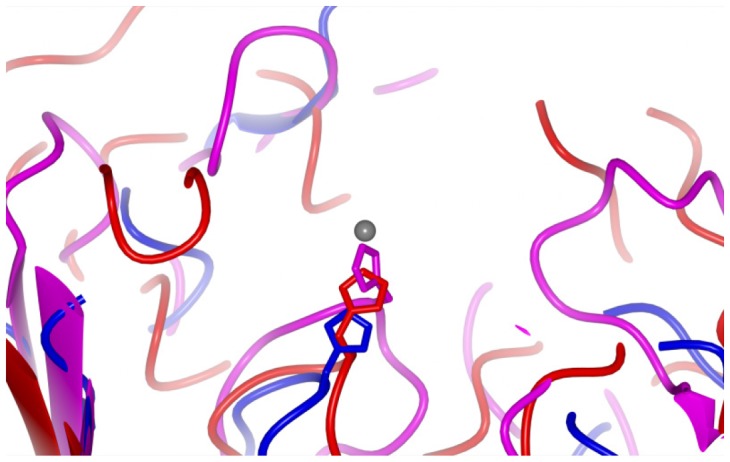
Comparison of FrpB with heme transporter structures. Superposition of FrpB F5-1 (red), ShuA (PDB 3FHH; blue) and HasR (PDB 3DDR; magenta). ShuA and HasR were superimposed onto the FrpB F5-1 structure using the SSM superpose function within Coot [44].

Comparison of the FrpB structure with those of other TBDTs revealed an unanticipated similarity with two heme transporters, ShuA [35] and HasR [10]. The FrpB F3-3 structure was solved by molecular replacement using a search model based on ShuA, a choice based on output from the program BALBES [34], although sequence identity, at about 21%, is still low. Superposition of both the HasR [10] and ShuA [35] TonB-dependent heme transporters also indicated His residues from the plug domain in a similar position to His133, although the other iron coordination residues from FrpB are not conserved (Figure 5). A similar comparison of FrpB with the structure of FepA, an enterobactin TBDT from *E. coli* [57], gave a lower sequence identity of 14% and no histidine in that position. The FrpB structure therefore indicates a possible evolutionary relationship to the heme transporters, although it is difficult to establish this with certainty given the limited number of TonB-dependent heme transporter structures currently available.

### The structural conservation of the HR region and its independence from iron binding

**Figure 6:**
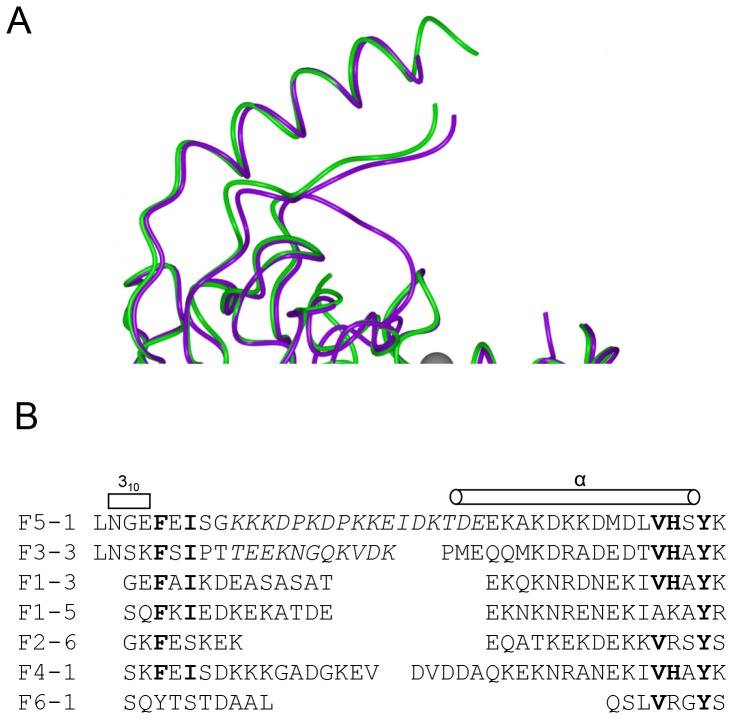
Structure and alignment of the FrpB hypervariable sequence regions. (A) Superposition of two hypervariable regions F5-1 purple, F3-3 (A chain) green. (B) Sequence alignment of the HR regions from the major FrpB variants.

A notable feature of the FrpB structure is the location of the hypervariable (HR) sequence region [24], which stands prominently above the rest of the protein and would be expected to protrude well above the membrane surface, where it would be accessible to antibody (Figure 1C). Despite substantial sequence differences, the HR region adopts a similar structure in both the F3-3 and F5-1 variants, consisting of a short helix at the N-terminus, followed by an extended strand which packs against a C-terminal helix (Figure 6A). Some residues were missing from the electron density at the tip for both variants, and were omitted from the final structure. One reason for the consistency of the structure of the antigenic region for both variants is the conservation of a group of residues at the base of the structure, which pack into a small hydrophobic core between the C-terminal helix and the loop, and consist of Phe399, Ile401, Val432, His433 and Y435 (F3-3 variant residue numbering). Alignment of this section of the sequence with representatives from the main families of other FrpB HR variants shows that these key residues are well conserved (Figure 6B). Secondary structure predictions corroborated the presence of an alpha helix at the C-terminus within other HR regions. Sequences from different FrpB variants are most variable at the tip of the structure, between the strand and the C-terminal helix. This would also correspond to the part of the structure which is furthest above the outer membrane, and hence most accessible to antibody.

**Figure 7:**
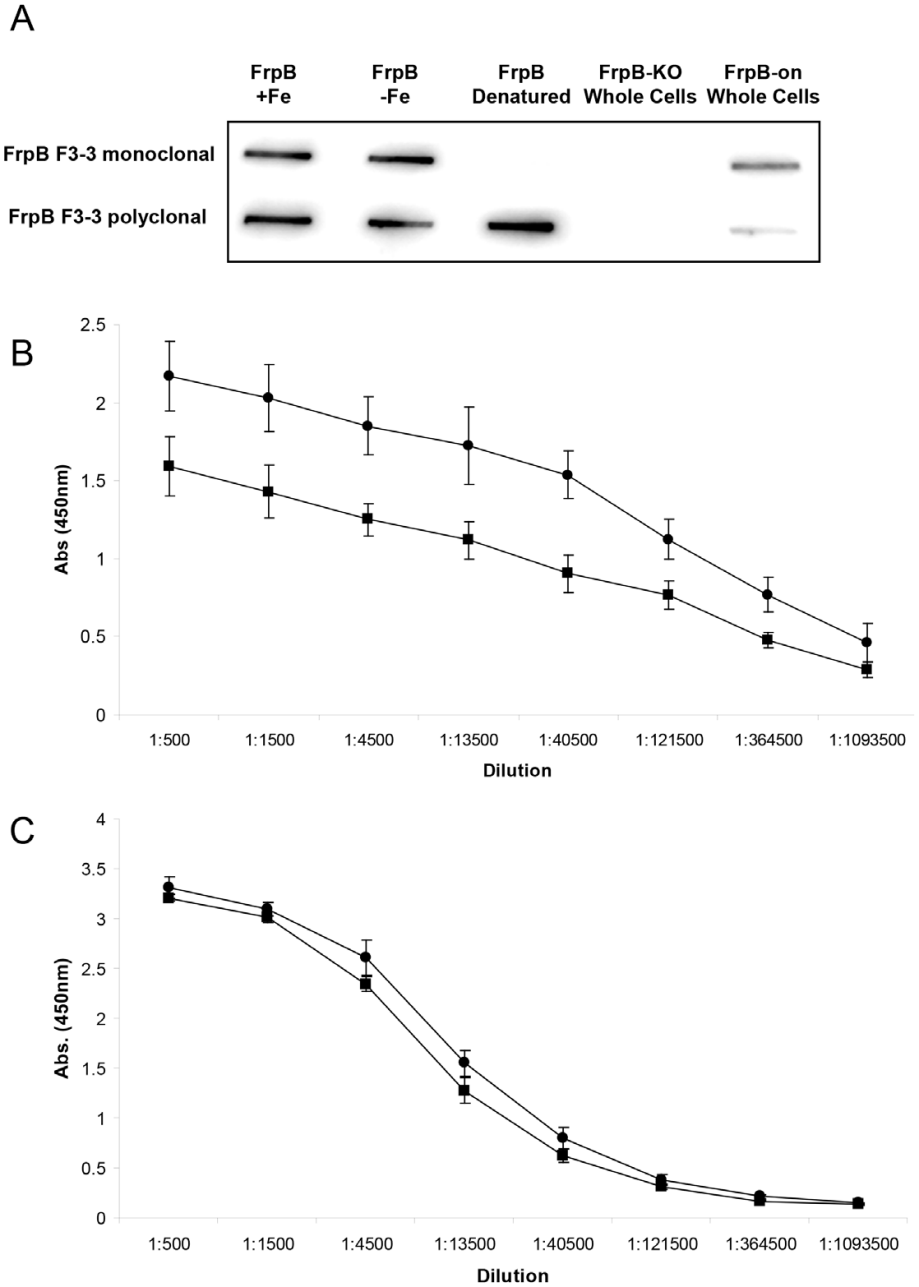
A comparison of the reactivity of antibody against FrpB F3-3 in the unbound and Fe^3+−^loaded states. (A) Slot blot (B) ELISA of reactivity of monoclonal antibody against FrpB F3-3 (•, +Fe; ▪, −Fe). Values are ± SD (n = 3). (C) ELISA of reactivity of polyclonal antibody against FrpB F3-3; symbols as (B). Values are ± SD (n = 3).

The separation between the antigenic loop, on the one hand, and the residues involved in iron recognition on the other, is a striking feature of the FrpB structure. Each seems to be independent of the other (the F3-3 and F5-1 variants showed no difference in iron binding affinity or capacity). To examine this question further, the reactivities of a monoclonal antibody specific for the FrpB F3-3 HR region and polyclonal antisera, also raised against FrpB F3-3, were examined against Fe-loaded and unloaded FrpB. A slot blot shows that recombinant FrpB reacts equally well with the monoclonal antibody or polyclonal antisera, irrespective of whether Fe^3+^ is bound to the protein or not (Figure 7A). The antibody also detects FrpB F3-3 in whole meningococci, but not in a *frpB* minus knock-out mutant or recombinant FrpB F3-3 which has been denatured. The results with the polyclonal antisera are similar, although reaction is also seen with denatured FrpB, and binding to whole meningococci is weaker. It is likely that many of the epitopes recognized by the polyclonal antibody are linear, and effectively masked when FrpB is in its native, membrane-embedded state in meningococci. ELISA assays confirmed that the reactivity of both the monoclonal antibody and polyclonal antisera were similar towards FrpB F3-3 in both its unloaded and iron-loaded states, with a small preference for Fe-FrpB (Figures 7B & C). Fe^3+^ binding therefore has relatively little effect on the recognition of FrpB by antibody, as anticipated from the F5-1 crystal structure.

## Discussion

There are multiple uptake pathways for iron into *Neisseria meningitidis*, reflecting the importance of iron acquisition to the organism. Iron is extracted from storage proteins, such as transferrin, ferrisiderophore, or else is taken up in the form of heme, which is removed from hemoglobin or other hemoproteins [18]. Transport of iron or heme across the outer membrane is mediated by specialized TBDTs, with defined specificities for each iron source. At present, the role of FrpB in iron uptake in *Neisseria* is less clear: it is well established that its expression is induced by iron privation [20] and binding studies have demonstrated an affinity for ferric enterobactin [22]. More recent work has extended its specificity to other, structurally related, catecholate-type siderophores [21]. The reported affinity of FrpB for ferric enterobactin is, however, weaker than has been observed for other TBDTs binding to iron-siderophores [6] and the multiple uptake pathways for iron in meningococci have made analysis of iron-dependent transport in *frpB* knockout mutants difficult. The observations reported here have provided strong evidence that recombinant FrpB binds to Fe^3+^ with high affinity and that the binding is to a single site, located in a similar position to transported substrates in other TBDT structures [6]. It is also apparent that binding of iron to FrpB induces structural changes, notably at the N-terminus close to the predicted location of the TonB box, which have also been observed on substrate binding by other TDBTs [6], [14]. Intriguingly, our data also show an increase in iron content when FrpB is incubated with ferric enterobactin, and abolition of this effect when two of the Fe-chelating residues are mutated (Figure 3). The explanation most consistent with these observations is that FrpB is capable of transporting both ferric enterobactin and the Fe^3+^ ion, depending on substrate availability. If this is the case it would constitute a remarkable degree of plasticity in substrate binding for a TBDT, but it needs to be demonstrated experimentally.

Levels of free Fe^3+^ in solution are low and, consequently, there is little apparent need for a transporter dedicated to the uptake of free Fe^3+^. Other iron uptake systems in *Neisseria* use sources of iron which are much more plentiful and to which the organism would have access *in vivo*. Nevertheless, our observations make a compelling argument for the biological relevance of Fe^3+^ binding to FrpB. The binding site is specific: it was, by some distance, the highest peak in the anomalous difference map after soaking and back-soaking of the FrpB F5-1 crystals with iron. FrpB must have a very high affinity for iron: we found that EPR spectra of some FrpB preparations showed traces of Fe^3+^ even without addition of exogenous iron, presumably acquired from solution after refolding. The identification of a distinct sub-family of TBDTs with the iron-chelating residues absolutely conserved also argues that the binding site is of physiological relevance (Figure S3). A solution to this conundrum could lie in observations made on the structure and binding specificity of Fbp, the periplasmic iron-binding protein. Although Fbp and FrpB adopt quite different tertiary structures, they both contain a dipeptide motif which plays a critical role in iron recognition: Tyr-Tyr in the case of Fbp [58], and Tyr-His in the case of FrpB. As well as binding mononuclear Fe^3+^, the open nature of the iron-binding site in Fbp allows it to bind to a range of multinuclear oxide or hydroxide-bridged iron species, through minor structural adaptations within the binding site [58], [59]. Multinuclear iron clusters can be present at much higher concentrations in solution than Fe(OH)_3_, and could be a viable source of iron to the bacterium. The binding of such clusters to FrpB remains to be formally demonstrated, but it is plausible that its specificity could extend beyond the binding of mononuclear iron, in an analogous fashion.

The location of the principal HR antigenic loop, which lies well above the other external loops in both crystal forms (Figure 1), agrees well with the observations of Kortekaas *et al.*, who provided evidence for the shielding of other immunogenic epitopes by this region [60]. It is striking that, in the alignment of TBDTs related to FrpB, the principal antigenic region is absent from other members of the sub-family (Figure S3), suggesting that it may have been acquired later in evolutionary time. Epidemiological modeling of variants of the PorA porin protein within meningococcal populations has established that the pattern of emergence of particular sequence variants, which persist for considerable periods of time, is consistent with a model where each variant is under strong immune selection [25], [26]. At the molecular level, the FrpB structures imply that this has resulted in the emergence of a specific structural feature which appears to have no function other than to interact with the immune system. Our data therefore show that sequence variation within the HR region is perhaps more complex than previously thought, in the sense that selection pressure has resulted in the conservation of a structural motif which is specifically associated with immune recognition and appears to have particular, conserved features which result in the formation of a scaffold onto which more variable sequences can be grafted. The need to preserve such a scaffold places constraints on the degree of sequence variability within the HR, in the form of conservation of key residues needed to maintain the scaffold structure. Furthermore, there is a preponderance of Asp, Glu, Lys and Asn residues in different sequence variants within the tip of the antigen motif, suggesting a selection bias towards hydrophilic side chains. The process of immune selection has therefore resulted in the emergence of sequence variants which are far from random but, rather, are determined by structural as well as immunological constraints.

This behavior forms a contrast with the vaccine component factor H binding protein, where variant residues are distributed over a much wider part of the protein surface [61]. This is understandable, as factor H binding protein is formed from two globular domains, and is not an integral membrane protein. It requires a much higher degree of exposure to mediate its function- binding to serum factor H- and hence a much wider range of the protein surface is accessible to antibody. The antigenic variability of FrpB is more characteristic of other integral OMPs, such as PorA, where sequence variation is concentrated into a few external surface loop regions [2]. The identification of a specific sub-domain in FrpB associated with antigenic variability has obvious ramifications for vaccine development, suggesting strategies for grafting the sub-domain on to other proteins, or engineering FrpB in such a way as to remove its transport activity but retain the antigenic sub-domain. This latter approach could assist in the development of attenuated *N. meningitidis* strains which will have applications in vaccine manufacture.

## Supporting Information

Figure S1 **Effect of Fe^3+^ titration on EPR Cu^2+^ spectra of FrpB.** 100 µM CuSO_4_ was added to 150 µM FrpB, before dialysis to remove unbound ions a subsequent addition of 0, 100, 200 and 300 µM FeCl_3_ (for 0, 1, 2 and 3-fold molar excess of Fe^3+^ over Cu^2+^) and collection of EPR spectra. Spectra for the FrpB F3-3 variant are on the left, and for the F5-1 variant on the right.
(TIF)

Figure S2 **Effect of Fe^3+^ titration on EPR Cu^2+^ spectra of FrpB F3-3 H133A.** 100 µM CuSO_4_ with 0, 100, 200 and 300 µM FeCl_3_ (for 0, 1, 2 and 3-fold molar excess of Fe^3+^ over Cu^2+^) was added to 150 µM FrpB, before dialysis to remove unbound ions and collection of EPR spectra.
(TIF)

Figure S3 **Sequence alignment of FrpB with orthologs from other Gram-negative bacteria.** The alignment was carried out using Clustalx [62], with some manual adjustment. Residues involved in coordinating the Fe are highlighted in cyan and the HR sequence region is highlighted in yellow.
(TIF)
