# Supplementary figures and images for: Use of a Molecular Decoy to Segregate Transport from Antigenicity in the FrpB Iron Transporter from Neisseria meningitidis
Source: PLoS One. 2013 Feb 15;8(2):e56746. doi: 10.1371/journal.pone.0056746 (PMC3574120; doi:10.1371/journal.pone.0056746)

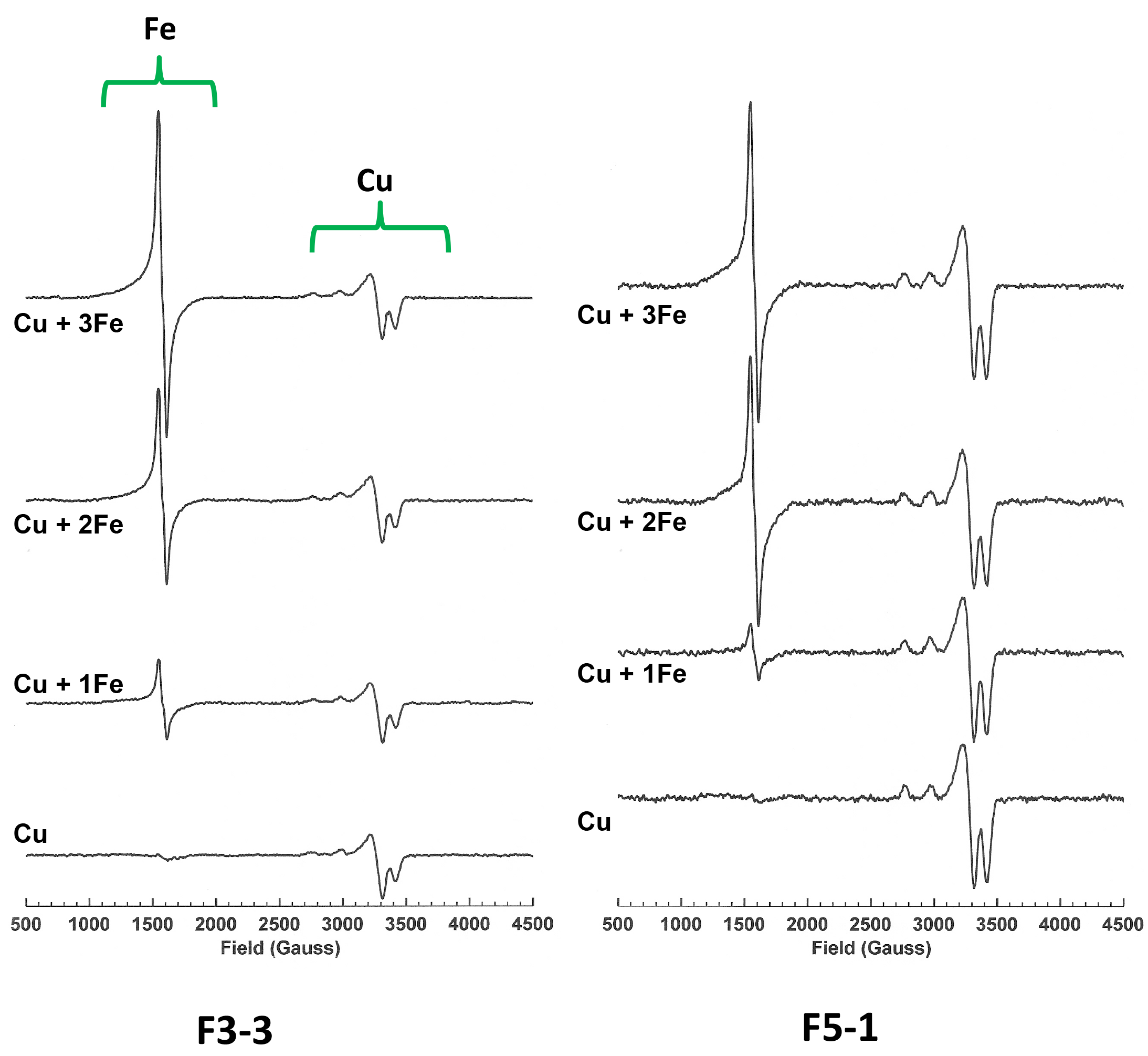

Supplement: Figure S1 — Effect of Fe3+ titration on EPR Cu2+ spectra of FrpB. 100 µM CuSO4 was added to 150 µM FrpB, before dialysis to remove unbound ions a subsequent addition of 0, 100, 200 and 300 µM FeCl3 (for 0, 1, 2 and 3-fold molar excess of Fe3+ over Cu2+) and collection of EPR spectra. Spectra for the FrpB F3-3 variant are on the left, and for the F5-1 variant on the right. (TIF) [file pone.0056746.s001.tif]

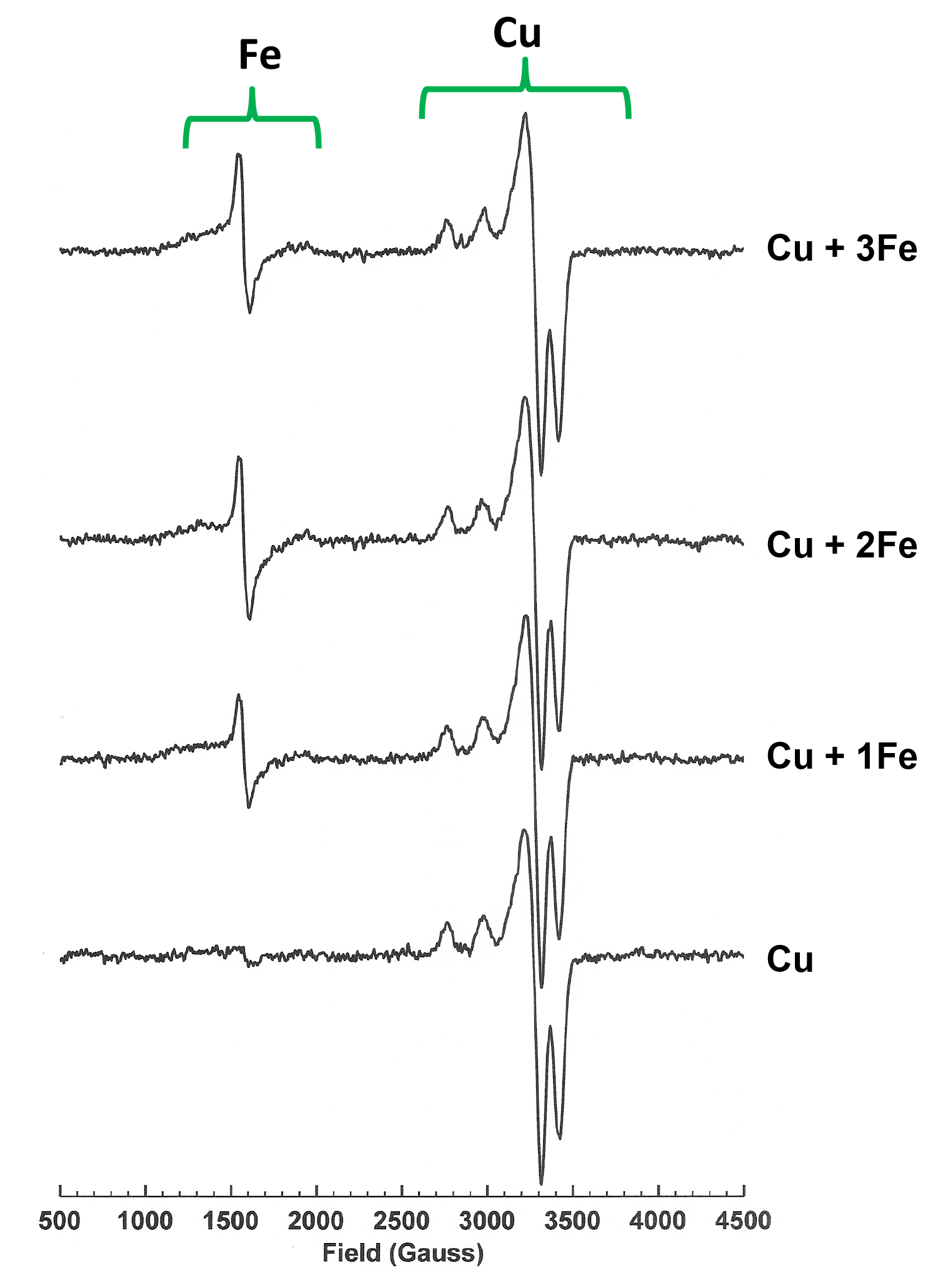

Supplement: Figure S2 — Effect of Fe3+ titration on EPR Cu2+ spectra of FrpB F3-3 H133A. 100 µM CuSO4 with 0, 100, 200 and 300 µM FeCl3 (for 0, 1, 2 and 3-fold molar excess of Fe3+ over Cu2+) was added to 150 µM FrpB, before dialysis to remove unbound ions and collection of EPR spectra. (TIF) [file pone.0056746.s002.tif]

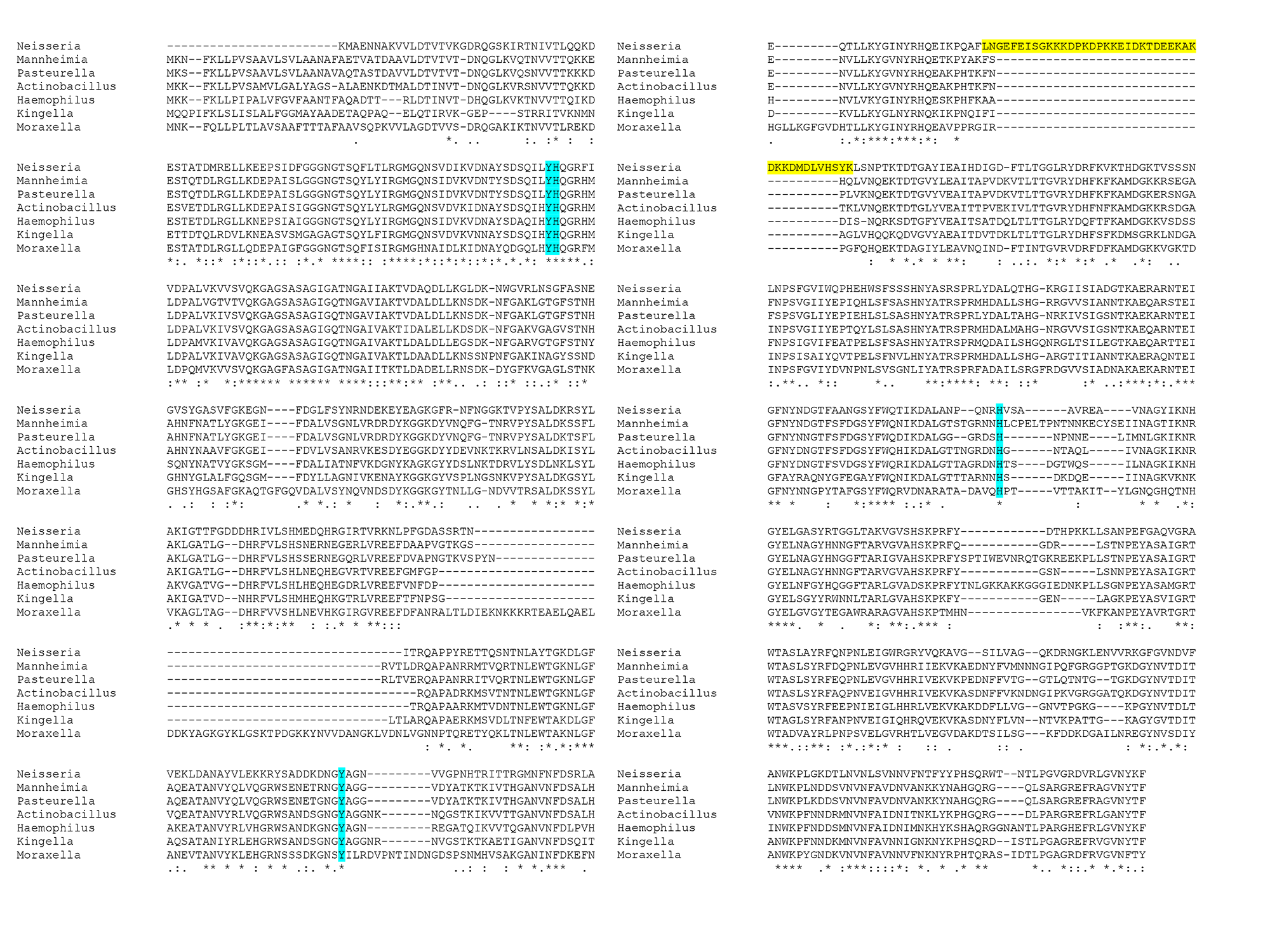

Supplement: Figure S3 — Sequence alignment of FrpB with orthologs from other Gram-negative bacteria. The alignment was carried out using Clustalx [62], with some manual adjustment. Residues involved in coordinating the Fe are highlighted in cyan and the HR sequence region is highlighted in yellow. (TIF) [file pone.0056746.s003.tif]
